# Comparative Efficacy of Intranasal Corticosteroids and Antihistamines in Enhancing Paediatric Quality of Life in Allergic Rhinoconjunctivitis

**DOI:** 10.1111/cea.70360

**Published:** 2026-06-03

**Authors:** Ellen Tameeris, Suzie J. van den Berg, Arthur M. Bohnen, Patrick J. E. Bindels, Gijs Elshout

**Affiliations:** ^1^ Department of General Practice Erasmus Medical Center Rotterdam Rotterdam the Netherlands

## Abstract

- Daily intranasal corticosteroids improved quality of life most in adolescents (12–18 years) with seasonal allergic rhinoconjunctivitis.
- Antihistamines showed greater quality‐of‐life improvement than intranasal corticosteroids in children (6–11 years).

To the Editor,

1

Allergic rhinoconjunctivitis (AR) is highly prevalent in children and adolescents and is associated with impaired quality of life (QoL), reduced sleep, affected school performance and daily activities [1]. Intranasal corticosteroids (INCS) and antihistamines (AH) are recommended first‐line therapies, yet evidence comparing their effects on QoL in paediatric patients is limited. Current guidelines emphasise QoL as the most relevant outcome in AR, but few randomised studies have evaluated QoL differences between treatment regimens in children and adolescents separately [2].

We conducted a post hoc analysis of a single‐blinded, three‐armed randomised controlled trial assessing symptomatic treatment for pollen‐induced AR in primary care. The original trial demonstrated no differences between treatment strategies in the percentage of symptom‐free days [3]. In the present analysis, we evaluated changes in QoL across treatment regimens over an entire pollen season.

Children and adolescents aged 6–18 years with seasonal AR due to grass (± birch) pollen were randomized to one of three treatment arms for three months during the grass pollen season: (1) Daily INCS (fluticasone propionate nasal spray 100 μg/day for children < 12 years and 200 μg/day for adolescents ≥ 12 years); (2) On‐demand INCS; and (3) On‐demand oral AH (Levocetirizine 5 mg).

QoL was assessed at baseline (pre‐season), midpoint (1–2 months) and endpoint (3 months) using validated questionnaires: the Paediatric Rhinoconjunctivitis Quality of Life Questionnaire (PRQLQ; ages 6–11) and the Adolescent Rhinoconjunctivitis Quality of Life Questionnaire (AdolRQLQ; ages 12–18) [4]. A change of 0.5 points represents the minimal clinically important difference (MCID) [5]. Additional study methods, including full study protocol, detailed methods and results are available in the Open Science Framework online repository: https://osf.io/jd4pf/overview?view_only=3d09e281ef7b476683c532ce8b022b7a.

Of the 150 randomized participants, 132 completed all QoL assessments and were included in this analysis (62 children, age 6–11 years and 70 adolescents, age 12–18 years). Baseline QoL scores were low across all treatment arms, reflecting relatively good QoL prior to pollen exposure. There were no clinically relevant baseline differences between treatment groups in either age category.

Daily INCS was compared to INCS on‐demand and to AH in improving QoL from baseline to midpoint and endpoint, using ANCOVA to adjust for gender and age.

In adolescents, daily INCS resulted in a statistically significant and clinically relevant improvement in overall QoL from baseline to endpoint (mean change −0.55), exceeding the MCID. Improvements were most pronounced in nasal and practical domains. In contrast, adolescents using on‐demand INCS or AH experienced a transient worsening of QoL during the peak of the pollen season, with no clinically relevant improvement by season's end. Daily INCS use was superior to on‐demand INCS in change from baseline for total, nasal and practical domain scores (Table 1).

**TABLE 1:** PRQLQ and AdolRQLQ at baseline, midpoint and endpoint.

	Treatment	Baseline (Mean, SD)	Midpoint (Mean, SD)	Endpoint (Mean, SD)	Change to midpoint (Mean (SD))	Change to endpoint (Mean (SD))
PRQLQ	INCS daily	1.39 (0.85)	1.32 (0.70)	1.44 (0.80)	−0.08 (0.75)	0.05 (1.11)
	INCS on‐demand	1.49 (1.49)	1.69 (1.11)	1.04 (0.87)	0.20 (1.18)	−0.45 (1.50)
	AH	1.85 (0.89)	1.48 (0.73)	1.09 (0.85)	−0.36 (1.15)	−0.76^a^ ^,^ ^b^ (1.13)
ADOLRQLQ	INCS daily	1.84 (1.09)	1.33 (0.84)	1.29 (0.99)	−0.51^a^ (1.01)	−0.55^a^ (1.27)
	INCS on‐demand	1.45 (1.08)	1.78 (1.03)	1.36 (1.10)	0.33 (1.17)	−0.09^b^ (0.90)
	AH	1.58 (1.17)	1.88 (0.89)	1.41 (0.97)	0.31 (0.82)	−0.17 (1.20)

^a^ *p*‐value < 0.05 for change from baseline, MCID = 0.5.

^b^ *p*‐value < 0.05 compared to INCS daily (ANCOVA analysis).

In children, AH use was associated with a clinically relevant and statistically significant improvement in overall QoL from baseline to endpoint (mean change −0.76). Improvements were most evident in the practical and activity symptom domains. Neither daily nor on‐demand INCS achieved clinically meaningful QoL improvement in this age group (Table 1). Compared with daily INCS, AH resulted in significantly greater improvement in the practical domain.

Medication adherence differed by age. Children used a higher proportion of prescribed INCS than adolescents, whereas on‐demand INCS use occurred on fewer than one‐third of study days overall, particularly among adolescents. These differences may have contributed to the observed age‐specific effects.

Our findings highlight clinically relevant differences between children and adolescents in QoL responses to AR treatment. Adolescents appeared to benefit most from daily INCS, likely reflecting a greater impact of nasal symptoms and functional limitations in this age group. In contrast, children showed greater improvement in everyday functioning with AH treatment, particularly regarding practical aspects.

Despite observable improvements, absolute differences in QoL between treatment groups at the end of the pollen season were small. Baseline QoL scores were already low, limiting potential improvement. Nevertheless, clinically meaningful changes were detectable over the season, even in a largely well‐controlled primary care population.

Strengths of this study include follow‐up over a complete pollen season and separate analyses for children and adolescents. Limitations include moderate sample sizes for subgroup analyses and the post hoc nature of the QoL evaluation.

In conclusion, daily INCS produced the greatest improvement in QoL among adolescents with seasonal AR, while AH was associated with superior QoL improvement in children. Given the modest differences between treatments and concerns regarding cumulative corticosteroid exposure, treatment decisions should be individualized, incorporating symptom profile, pollen exposure, age, adherence, potential adverse effects, effects on symptoms and QoL and shared decision‐making.

## Author Contributions

**Ellen Tameeris:** study design, data management, statistical analysis, data interpretation, manuscript writing. **Suzie J. van den Berg:** statistical analysis, manuscript writing, data interpretation. **Arthur M. Bohnen:** data collection, study design (supervision), statistical analysis (supervision), data interpretation, manuscript writing (supervision). **Patrick J. E. Bindels:** data interpretation (supervision), manuscript writing (supervision). **Gijs Elshout:** data interpretation (supervision), manuscript writing (supervision).

## Funding

The authors have nothing to report.

## Conflicts of Interest

The authors declare no conflicts of interest.

## Data Availability

The data that support the findings of this study are available on request from the corresponding author. The data are not publicly available due to privacy or ethical restrictions. However, full tables and extended results of the study are available in a data repository.
